# Genomic Variations in SARS-CoV-2 Genomes From Gujarat: Underlying Role of Variants in Disease Epidemiology

**DOI:** 10.3389/fgene.2021.586569

**Published:** 2021-03-19

**Authors:** Madhvi Joshi, Apurvasinh Puvar, Dinesh Kumar, Afzal Ansari, Maharshi Pandya, Janvi Raval, Zarna Patel, Pinal Trivedi, Monika Gandhi, Labdhi Pandya, Komal Patel, Nitin Savaliya, Snehal Bagatharia, Sachin Kumar, Chaitanya Joshi

**Affiliations:** ^1^Gujarat Biotechnology Research Centre (GBRC), Department of Science & Technology (DST), Gandhinagar, India; ^2^Gujarat State Biotechnology Mission, Gandhinagar, India; ^3^Indian Institute of Technology Guwahati, Guwahati, India

**Keywords:** genomic surveillance, mutation analysis, SARS-CoV-2 (2019-nCoV), COVID-19, viral epidemiology, haplotyping

## Abstract

Humanity has seen numerous pandemics during its course of evolution. The list includes several incidents from the past, such as measles, Ebola, severe acute respiratory syndrome (SARS), and Middle East respiratory syndrome (MERS), etc. The latest edition to this is coronavirus disease 2019 (COVID-19), caused by the novel coronavirus, severe acute respiratory syndrome coronavirus 2 (SARS-CoV-2). As of August 18, 2020, COVID-19 has affected over 21 million people from 180 + countries with 0.7 million deaths across the globe. Genomic technologies have enabled us to understand the genomic constitution of pathogens, their virulence, evolution, and rate of mutation, etc. To date, more than 83,000 viral genomes have been deposited in public repositories, such as GISAID and NCBI. While we are writing this, India is the third most affected country by COVID-19, with 2.7 million cases and > 53,000 deaths. Gujarat is the 11th highest affected state with a 3.48% death rate compared to the national average of 1.91%. In this study, a total of 502 SARS-CoV-2 genomes from Gujarat were sequenced and analyzed to understand its phylogenetic distribution and variants against global and national sequences. Further variants were analyzed from diseased and recovered patients from Gujarat and the world to understand its role in pathogenesis. Among the missense mutations present in the Gujarat SARS-CoV-2 genomes, C28854T (Ser194Leu) had an allele frequency of 47.62 and 7.25% in deceased patients from the Gujarat and global datasets, respectively. In contrast, the allele frequency of 35.16 and 3.20% was observed in recovered patients from the Gujarat and global datasets, respectively. It is a deleterious mutation present in the nucleocapsid (N) gene and is significantly associated with mortality in Gujarat patients with a *p*-value of 0.067 and in the global dataset with a *p*-value of 0.000924. The other deleterious variant identified in deceased patients from Gujarat (*p*-value of 0.355) and the world (*p*-value of 2.43E-06) is G25563T, which is located in Orf3a and plays a potential role in viral pathogenesis. SARS-CoV-2 genomes from Gujarat are forming distinct clusters under the GH clade of GISAID. This study will shed light on the viral haplotype in SARS-CoV-2 samples from Gujarat, India.

## Introduction

As per the recent situation report-209 released by the World Health Organisation (WHO), as accessed on August 18, 2020, the total confirmed positive cases of COVID-19 across the globe are 21,294,845 resulting in 761,779 deaths. In many countries, such as China, Spain, Australia, Japan, South Korea, and the United States, the second wave of severe acute respiratory syndrome coronavirus 2 (SARS-CoV-2) infections has started (Evenett and Winters, 2020; Leung et al., 2020; Strzelecki, 2020; Xu and Li, 2020). India is the third most affected country by coronavirus disease 2019 (COVID-19) after the United States and Brazil, with 2,771,958 cases and 53,046 deaths, respectively. Gujarat is located in the western part of India. It is the 11th highest affected state in India, with 80,942 cases and 2,820 deaths as per the https://www.covid19india.org/. However, the death rate in the state of Gujarat is 3.48% with a recovery rate of 78.83%, which is 5% higher than the existing recovery rate in India. Therefore, understanding the pathogen evolution and virulence through genome sequencing will be key to understanding its diversity, variation, and its effect on pathogenesis and disease severity. Global repositories, such as the GISAID and NCBI databases, are flooded with SARS-CoV-2 genomes with an average of 381 genomes per day being added from across the globe. SARS-CoV-2 genome size is 29–30.6 kb. The genome includes 10 genes that encode 4 structural and 16 non-structural proteins (NSPs). Structural proteins are encoded by the four structural genes, including spike (S), envelope (E), membrane (M), and nucleocapsid (N) genes. The ORF1ab is the largest gene in SARS-CoV-2, which encodes the pp1ab protein and 15 NSPs. The ORF1a gene encodes for pp1a protein, which also contains 10 NSPs (Du et al., 2009; Shereen et al., 2020).

In the present study, the whole genome of 502 SARS-CoV-2 from Gujarat has been sequenced and analyzed against 2,121 SARS-CoV-2 genomes across the globe with known patient status. The overall dataset comprises 361 confirmed positive COVID-19 patients, which included 122 female and 239 male patients from Gujarat, India. Furthermore, a total of 502 viral genomes were sequenced from 361 samples based on the dominant and recessive allelic frequencies. These genomes were studied against a total of 79,518 complete viral genome sequences as accessed on August 18, 2020 to characterize their clades and variant distribution. Further statistical tools were applied to understand the differences in the variants with respect to disease epidemiology. In the absence of clinically approved drugs and other possible therapies in treating COVID-19, tracking pathogen evolution through whole genome sequencing is instrumental in understanding the progression of the pandemic locally as well as globally. This will further help in devising better strategies for vaccine development, identifying potential drug targets, and understanding host–pathogen interactions.

## Materials and Methods

### Sample Collection and Processing

Nasopharyngeal and oropharyngeal swabs from a total of 361 individuals who tested positive for COVID-19 from 46 locations representing 20 districts of Gujarat were collected after obtaining informed consent and appropriate ethics approval. The numbers of samples from these locations were selected on the basis of disease spread and incidence rate in Gujarat. The details of samples collected from each location are shown in Supplementary Table 1. Samples were transported as per standard operating procedures as prescribed by the World Health Organisation (WHO) and Indian Council of Medical Research (ICMR, New Delhi; SoP No. ICMR-NIV/2019-nCoV/Specimens_01) to a research laboratory and further stored at −20°C till processed.

### Whole Genome Sequencing of SARS-CoV-2

Total genomic RNA from the samples was isolated using the QIAamp Viral RNA Mini Kit (Cat. No. 52904; Qiagen, Germany) following the prescribed biosafety procedure. cDNA from the extracted RNA was made using the SuperScript^TM^ III Reverse Transcriptase first strand kit (Cat. No. 18080093; Thermo Fisher Scientific, United States) as per the procedures prescribed. SARS-CoV-2 genome was amplified by using the Ion AmpliSeq SARS-CoV-2 Research Panel (Thermo Fisher Scientific, United States) that consists of two pools with amplicons ranging from 125 to 275 bp in length and covering >99% of the SARS-CoV-2 genome, including all serotypes. Amplicon libraries were prepared using the Ion AmpliSeq^TM^ Library Kit Plus (Cat. No. A35907; Thermo Fisher Scientific, United States). These libraries were quantified using the Ion Library TaqMan^TM^ Quantitation Kit (Cat. No. 4468802; Thermo Fisher Scientific, United States). The quality of the library was checked using the DNA high sensitivity assay kit on Bio-analyser 2100 (Agilent Technologies, United States) and was sequenced on the Ion S5 Plus sequencing platform using a 530 chip.

### Raw Data Quality Assessment and Filtering

The quality of data was assessed using the FASTQC v. 0.11.5 (Andrews, 2010) toolkit. All raw data sequences were processed using the PRINSEQ-lite v.0.20.4 (Schmieder and Edwards, 2011) program for filtering the data. All sequences were trimmed from the right to where the average quality of 5 bp window was lower than QV25, 5 bp from the left end was trimmed, and sequences with length lower than 50 bp and sequences with average quality QV25 were removed.

### Genome Assembly, Variant Calling, and Global Dataset

Quality filtered data were assembled using reference-based mapping with CLC Genomics Workbench 12. Mapping was done using stringent parameters with a length fraction of 0.99 and a similarity fraction of 0.9. Mapping tracks were used to call and annotate variants. Variants were called using Ion Torrent variant caller with a minimum allele frequency of 30% with a minimum coverage of 10 reads considered. For comparative analysis with the global dataset, 79,518 complete viral genomes and 1,821 viral genome isolates from India were downloaded from the GISAID flu server^1^. Considering haplotypes (a, b) based on allelic frequency, a total of 502 genomes were sequenced from a total of 361 patients as mentioned in Supplementary Table 2.

### Phylogenetic Analysis

A total of 502 SARS-CoV-2 whole genomes were sequenced and analyzed for their phylogenetic distribution at different locations from Gujarat. The reference genome, Wuhan/Hu-1/2019 (EPI_ISL_402125), sampled on December 31, 2019, from Wuhan, China was downloaded from the GISAID flu server. Additionally, three viral genomes from the seafood market from Wuhan, China were included in the phylogenetic analysis (EPI_ISL_406798, EPI_ISL_402124, and EPI_ISL_406801). The multiple sequence alignment was performed using MAFFT (Katoh and Standley, 2013) implemented *via* a phylodynamic analysis pipeline provided by Augur^2^. The subsequent alignment output files were checked, visualized, and verified using PhyloSuite (Zhang et al., 2020). Afterward, the maximum likelihood phylogenetic tree was built using the Augur tree implementation pipeline with the IQ-TREE 2 (Minh et al., 2020) with default parameters. The selected metadata information plotted in the time-resolved phylogenetic tree was constructed using TreeTime (Sagulenko et al., 2018) and annotated and visualized in the FigTree (Rambaut, 2018).

### Statistical Analysis

The non-parametric chi-square test of significance was used to check the difference of variables, such as the effect on age, gender, and mutations on mortality for the Gujarat, India, and global datasets for the deceased versus recovered patients.

## Results

**FIGURE 1:**
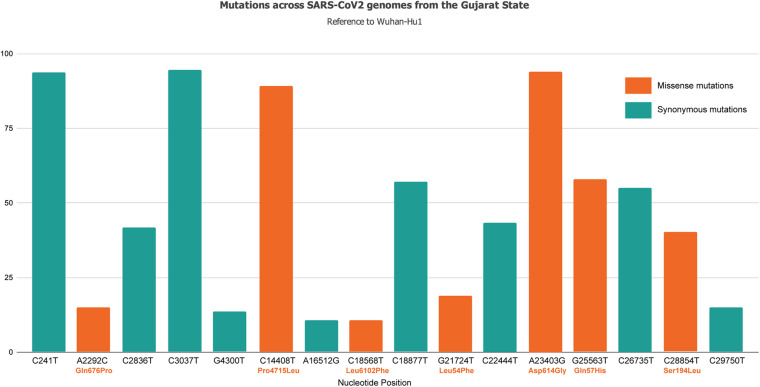
Mutation spectrum profile of 502 SARS-CoV-2 genomes from 46 locations representing 20 districts of Gujarat, India including synonymous and missense mutations. The top mutations included C241T, C3037T, C14408T/Pro314Leu, C18877T, A23403G/Asp614Gly, G25563T/Gln57His, and C26735T with frequency >55%.

Samples were collected based on COVID-19 incidence rate across Gujarat from 16 different originating laboratories representing 46 different geographical locations from 20 districts of Gujarat, India as mentioned in Supplementary Table 1. The geographical distributions of the top three locations of viral isolates are represented by Ahmedabad (*n* = 172), Vadodara (*n* = 92), and Surat (*n* = 86), respectively. A total of 502 viral genomes from 361 patients have been sequenced in the study from which 122 were from females, whereas 239 were from males. These patients were from 1 to 86 years old group with an average age of 47.91 years. Most of the COVID-19 positive patients had symptoms of fever, diarrhea, cough, and breathing problems, whereas some of them had comorbid conditions, such as hypertension, diabetes, etc. The final outcomes of these patients were classified as deceased, recovered, hospitalized, or unknown status for further data analysis based on the available metadata. These details are presented in Supplementary Table 2. Chi-square test was performed to test the effect of gender and age group for the Gujarat and global datasets. The female patients (at *p*-value of 2.7E-08) in the Gujarat dataset were observed to be at a significantly higher death rate than those in the global dataset in deceased and recovered patients. The genomic dataset was further divided into different age groups of up to 40, 41–60, and over 60 years. The results indicated a significantly higher mortality rate at the age groups of 41–60 (at *p*-value of 0.03783) and over 60 years in the Gujarat dataset (at *p*-value of 0.2084) than at the age groups in the global dataset. Life expectancy in India is 68.7 years as per the National Health Profile 2019 report released by the Central Bureau of Health Intelligence (CBHI), Ministry of Health and Family Welfare, Government of India. The mutation frequency profile of the Gujarat genome with the mutation spectrum is highlighted in Figure 1 including synonymous and missense mutations.

### Genome Sequencing and Haplotyping

Out of 361 patients, 141 had mixed infections. Mixed infections were judged by the frequency of heterozygous mutations. The heterozygous mutation was considered only if it was supported by forward and reverse reads of an amplicon. Genomes were observed for heterozygous allele frequencies and were manually divided into two genomes. As a result, from 141 patients, a total of 282 viral genomes were classified as two different haplotypes and annotated with suffixes “a” and “b.” All major alleles having read frequency ranging from 60 to 80% were included in the “a” haplotype, whereas minor alleles having read frequency ranging from 20 to 40% were included in the “b” haplotype as provided in Supplementary Table 2.

### Phylogenetic Analysis

**FIGURE 2:**
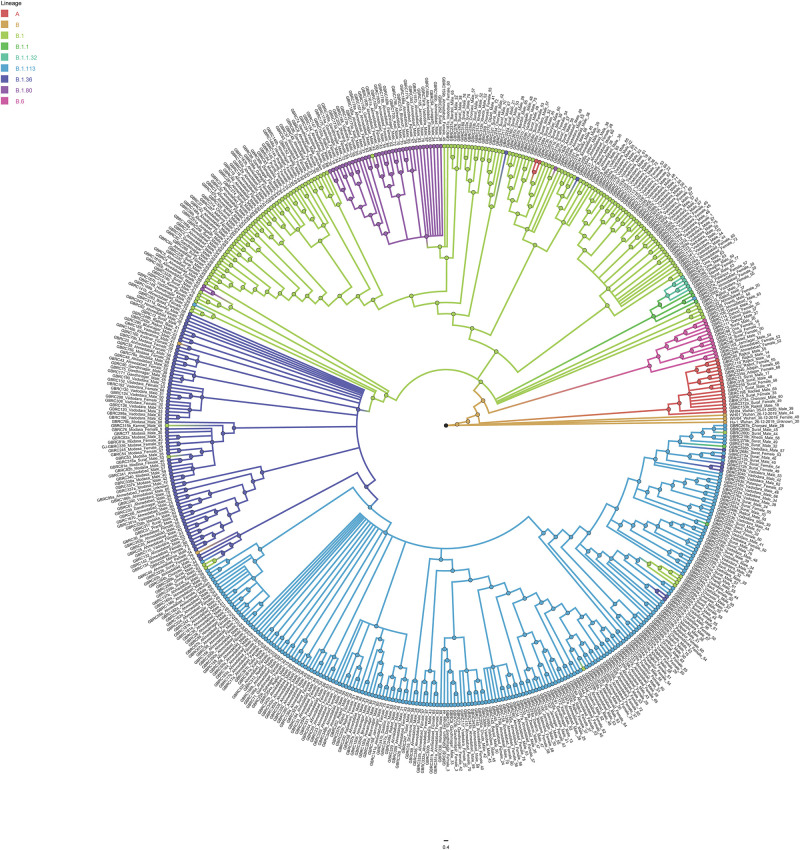
Phylogenetic distribution of lineage from 502 SARS-CoV-2 viral genomes from Gujarat, India with reference to the Wuhan/Hu-1/2019 (EPI_ISL_402125). Maximum likelihood phylogenetic tree was built using the Augur tree implementation pipeline with the IQ-TREE 2 with default parameters. The selected metadata information plotted in the time-resolved phylogenetic tree was constructed using TreeTime and visualized in the FigTree.

**FIGURE 3:**
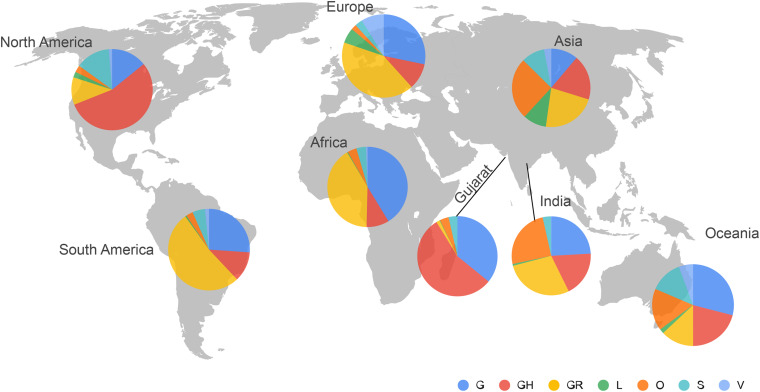
Distribution of the GISAID clades of SARS-CoV-2 genomes from global and Gujarat datasets as of 18th August 2020. The majority of the viral genomes from Gujarat are falling under GH (*n* = 278) and G (*n* = 180) clades.

Phylogenetic analysis of 502 genomes was done as per the definitions of the PANGOLIN lineage and GISAID clades. The overall lineages distribution highlighted the dominant occurrence of B.1.36 (*n* = 214), B.1 (*n* = 182), A (*n* = 18), B.6 (*n* = 12), B.1.1 (*n* = 9), and B (*n* = 4), whereas clade distribution highlighted the dominant prevalence of GH (*n* = 278), G (*n* = 180), O (*n* = 18), S (*n* = 18), GR (*n* = 7), and L (*n* = 1) as mentioned in Supplementary Table 3. While none of the genomes from Gujarat belonged to clade V, in the global perspective, the distribution of the GISAID clades as of 18th August 2020 from viral genome sequences indicates the dominance of GR clade (32.14%), G clade (23.72%), GH clade (22.66%), S clade (6.73%), L clade (5.13%), V clade (6.17%), and O clade (3.45%). The maximum likelihood time-resolved phylogeny tree in Figure 2 was constructed using the TreeTime pipeline and Augur bioinformatics pipeline and annotated and visualized in the FigTree (Hadfield et al., 2018; Rambaut, 2018; Mercatelli and Giorgi, 2020). Similarly, genomes classified into GISAID clades across the globe and Gujarat are highlighted in Figure 3.

### Comparative Analysis of Mutation Profile in SARS-CoV-2 Genomes

**FIGURE 4:**
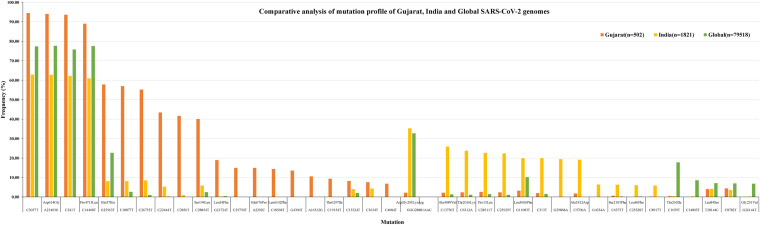
Synonymous and missense mutation profiles of the SARS-CoV-2 viral genomes, Gujarat (*n* = 502), India (*n* = 1,821), and global (*n* = 79,518). Only mutations with frequency >5% are plotted.

To understand the significance of the mutations in the SARS-CoV-2 genome isolates from the Gujarat, India, and global dataset, we have analyzed and compared the mutation profile of the 502 viral isolates from Gujarat along with the global dataset of 79,518 viral genomes and 1,821 viral genomes from India obtained from the GISAID server. A total of 27,455 mutations were observed in the global viral genome sequences (*n* = 79,518) of SARS-CoV-2 from GISAID wherein 3,478 mutations were observed from viral genomes from the Indian isolates (*n* = 1,821), whereas 752 mutations were observed in genomes sequenced from the Gujarat isolates (*n* = 502). Out of these mutations, 111 mutations were novel to viral isolates from the Gujarat genomes, and 1,164 were novel to the Indian genomes. The bar chart displaying the comparative mutation analysis is represented as Figure 4, with a frequency of >5% from the global, Indian, and Gujarat viral genomes including missense and synonymous mutations.

**FIGURE 5:**
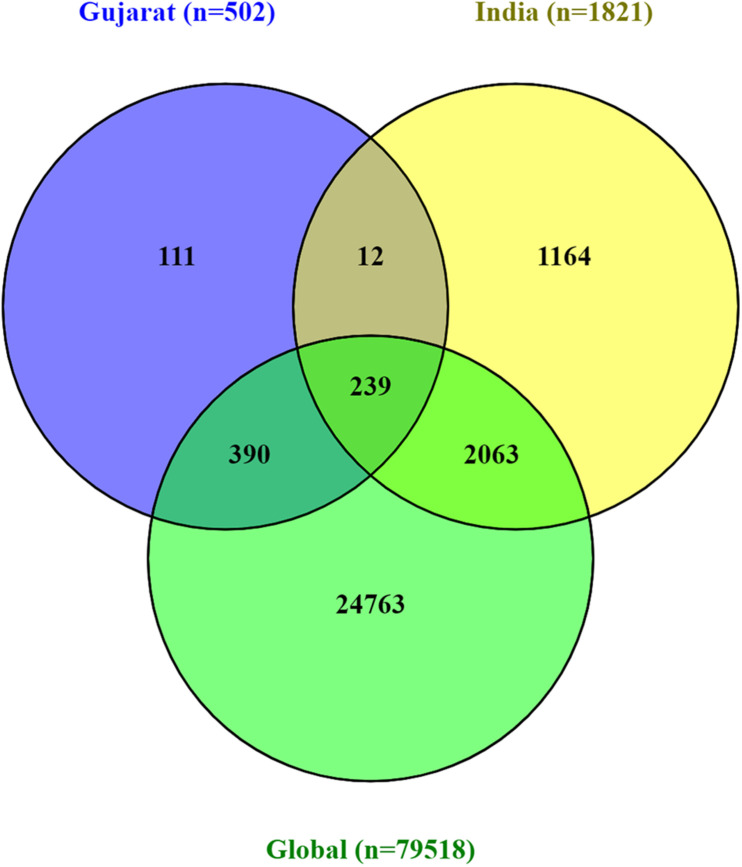
Venn diagram representing the mutually common and exclusive synonymous and missense mutations among SARS-CoV-2 viral genomes, Gujarat (*n* = 502), India (*n* = 1,821), and global (*n* = 79,518).

A Venn diagram represents the overall mutations shared between viral genome sequences analyzed from the global, Indian, and Gujarat isolates as given in Figure 5. A comparison of the mutation profile analysis with *p*-value significance, Sorting Intolerant from Tolerant (SIFT) score functional effect, frequency >5%, and absolute count of the number of genomes with prevalence is represented in Table 1. Further, frequencies of all the mutations were calculated by subtracting variants of the Gujarat genomes from the Indian and global genomes with statistical significance.

**TABLE 1:** The overall comparison of missense 478 and synonymous mutation frequency profiles of Gujarat-502, India-1821, and Global-79518 datasets.

			**Genome count**			**Frequency**					
**Gene**	**NT position**	**AA position**	**Gujarat (*n* = 502)**	**India (*n* = 1,821)**	**Global (*n* = 79,518)**	**Gujarat**	**India**	**Global**	**SIFT score**	**Functional effect**	***p*-Value**
5’ UTR	C241T		470	1,133	60,265	93.63	62.22	75.79	#N/A	#N/A	1.23505E-58
ORF1ab	C313T		10	362	1,178	1.99	19.88	1.48	0.84	Benign/tolerated	0
	C1059T	Thr265Ile	2	7	14,114	0.40	0.38	17.75	0.03	Deleterious	3.3988E-104
	A2292C	Gln676Pro	75	0	0	14.94	0.00	0.00	0.05	Deleterious	0
	C2836T		209	17	21	41.63	0.93	0.03	0.17	Benign/tolerated	0
	C3037T		474	1,145	61,503	94.42	62.88	77.34	0.66	Benign/tolerated	3.45605E-65
	C3634T		38	78	26	7.57	4.28	0.03	0.40	Benign/tolerated	0
	C4084T		34	1	35	6.77	0.05	0.04	0.72	Benign/tolerated	0
	G4300T		68	1	41	13.55	0.05	0.05	0.84	Benign/tolerated	0
	G4354A		0	116	0	0.00	6.37	0.00	1.00	Benign/tolerated	0
	C5700A	Ala1812Asp	9	348	8	1.79	19.11	0.01	0.38	Benign/tolerated	0
	C6312A	Thr2016Lys	12	432	882	2.39	23.72	1.11	0.03	Deleterious	0
	C6573T	Ser2103Phe	3	114	206	0.60	6.26	0.26	0.36	Benign/tolerated	0
	C8782T		22	65	5,526	4.38	3.57	6.95	0.67	Benign/tolerated	1.08234E-08
	C8917T		1	107	90	0.20	5.88	0.11	1.00	Benign/tolerated	0
	G11083T	Leu3606Phe	16	362	8,060	3.19	19.88	10.14	0.01	Deleterious	1.98676E-46
	C13730T	Ala4489Val	11	471	1,034	2.19	25.86	1.30	0.00	Deleterious	0
	C14408T	Pro4715Leu	447	1,110	61,641	89.04	60.96	77.52	0.31	Benign/tolerated	9.93477E-70
	C14805T		0	5	6,799	0.00	0.27	8.55	1.00	Benign/tolerated	2.09768E-45
	C15324T		41	73	1,588	8.17	4.01	2.00	1.00	Benign/tolerated	2.2731E-28
	A16512G		53	0	13	10.56	0.00	0.02	1.00	Benign/tolerated	0
	C18568T	Leu6102Phe	72	1	50	14.34	0.05	0.06	0.01	Deleterious	0
	C18877T		286	147	2,075	56.97	8.07	2.61	1.00	Benign/tolerated	0
	C19154T	Thr6297Ile	47	0	5	9.36	0.00	0.01	0.21	Benign/tolerated	0
	A20268G		0	3	4,650	0.00	0.16	5.85	1.00	Benign/tolerated	1.27368E-30
S	G21724T	Leu54Phe	95	4	304	18.92	0.22	0.38	0.69	Benign/tolerated	0
	C22444T		218	96	201	43.43	5.27	0.25	1.00	Benign/tolerated	0
	A23403G	Asp614Gly	472	1,142	61,751	94.02	62.71	77.66	0.30	Benign/tolerated	2.08832E-67
	C23929T		12	408	858	2.39	22.41	1.08	1.00	Benign/tolerated	0
ORF3a	C25528T	Leu46Phe	0	110	194	0.00	6.04	0.24	0.00	Deleterious	0
	G25563T	Gln57His	290	147	18,045	57.77	8.07	22.69	0.00	Deleterious	1.1597E-125
	G26144T	Gly251Val	0	4	5,385	0.00	0.22	6.77	0.00	Deleterious	1.93496E-35
M	C26735T		277	154	797	55.18	8.46	1.00	1.00	Benign/tolerated	0
ORF8	T28144C	Leu84Ser	20	75	5,636	3.98	4.12	7.09	0.37	Benign/tolerated	1.70788E-07
N	C28311T	Pro13Leu	13	413	1,151	2.59	22.68	1.45	0.00	Deleterious	0
	C28854T	Ser194Leu	201	106	1,948	40.04	5.82	2.45	0.05	Deleterious	0
	GGG28881AAC	ArgGly203LysArg	11	642	26,021	2.19	35.25	32.72	0.00	Deleterious	5.3828E-48
3’ UTR	C29750T		75	0	42	14.94	0.00	0.05	#N/A	#N/A	0
	G29868A		0	353	42	0.00	19.38	0.05	#N/A	#N/A	0

**FIGURE 6:**
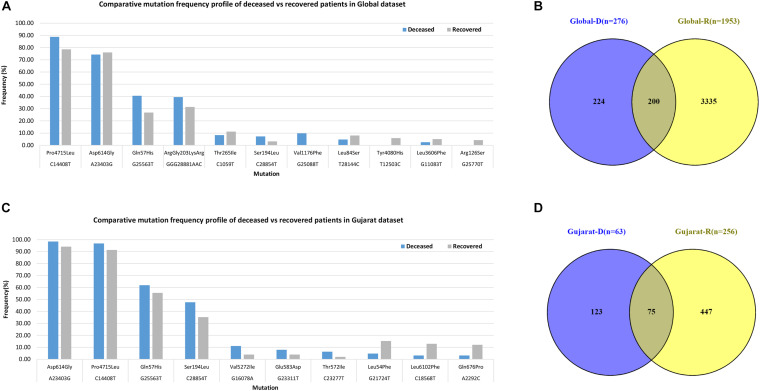
Frequency of missense mutations in SARS-CoV-2 viral genome from global dataset. **(A)** Bar chart for global deceased versus recovered patients. **(B)** Venn diagram of the global deceased versus recovered patients. **(C)** Bar chart for the Gujarat deceased versus recovered patients. **(D)** Venn diagram of the Gujarat deceased versus recovered patients.

Mutations (C241T, C3037T, A23403G, and C14408T) were dominant with frequency (>60%) in all the genomes (Gujarat, India, and global), whereas mutations (G11083T, C13730T, C28311T, C6312A, C313T, C5700A, G29868A, and C23929T) dominated (>19%) in the Indian genomes compared with the Gujarat and global genomes. The multi-nucleotide variant (MNV) GGG28881AAC is dominant in Indian (35.25%) and global genomes (32.72%), but in the context of Gujarat, it is present with a frequency of 2.19%. The mutations G25563T, C26735T, and C18877T (>55%), followed by C2836T, C22444T, and C28854T (>40%), followed by G21724T, C29750T, C18568T, G4300T, and A2292C (>13%) in viral genomes were sequenced from Gujarat. The detailed mutation frequency profile is provided as Supplementary Table 4. With reference to viral isolates from India, GGG28881AAC, G11083T, C28311T, C6312A, C23929T, and C13730T were found to be occurring at greater than 19% frequencies (*p*-value <0.001). Mutations G11083T and C6312A lie in the region of Orf1a encoding Nsp6, whereas mutation GGG28881AAC is present in the N gene. Further, deceased versus recovered patient mutation profile analysis of the known patient’s status dataset from Gujarat and global is represented in Figure 6 and Supplementary Tables 5, 6. Similarly, comparison of missense mutation profile of deceased versus recovered patients with genome count, frequency >5%, and *p*-value for the global dataset is represented in Table 2 and for the Gujarat dataset in Table 3; additionally, metadata for deceased and recovered patients is provided as Supplementary Tables 7, 8. The statistical significance association of gender and age of the deceased and recovered patients from the Gujarat and global dataset patients in both datasets was considered for analysis. Similarly, for age group 41–60 years, it highlighted the higher observation of death rate in patients with known status as given in Table 4.

**TABLE 2:** Comparison of missense mutation frequency in deceased 481 vs recovered patients from global dataset.

**NT mutation**	**AA mutation**	**Global mutation count (genomes)**		**Global frequency (%)**		**SIFT score**	**Functional effect**	***p*-Value**
		**Deceased (*n* = 276)**	**Recovered (*n* = 1,845)**	**Deceased**	**Recovered**			
C14408T	Pro4715Leu	245	1,450	88.77	78.59	0.31	Benign/tolerated	8.28E-05
A23403G	Asp614Gly	205	1,403	74.28	76.04	0.3	Benign/tolerated	0.522342
G25563T	Gln57His	112	495	40.58	26.83	0.00	Deleterious	2.43E-06
GGG28881AAC	ArgGly203LysArg	101	579	39.45	31.38	0.00	Deleterious	0.083557
C1059T	Thr265Ile	23	206	8.33	11.17	0.03	Deleterious	0.157376
C28854T	Ser194Leu	20	59	7.25	3.20	0.05	Deleterious	0.000924
G25088T	Val1176Phe	27	5	9.78	0.27	#N/A	#N/A	1.19E-33
T28144C	Leu84Ser	13	148	4.71	8.02	0.37	Benign/tolerated	0.052701
T12503C	Tyr4080His	0	109	0.00	5.91	0.00	Deleterious	3.38E-05
G11083T	Leu3606Phe	7	94	2.54	5.09	0.01	Deleterious	0.062656
G25770T	Arg126Ser	0	79	0.00	4.28	0.00	Deleterious	0.000459

**TABLE 3:** Comparison of missense mutation frequency in deceased 485 vs recovered patients from Gujarat dataset.

		**Gujarat mutation count (genomes)**		**Gujarat frequency (%)**				
**NT mutation**	**AA mutation**	**Deceased (*n* = 63)**	**Recovered (*n* = 256)**	**Deceased**	**Recovered**	**SIFT score**	**Functional effect**	***p*-Value**
A23403G	Asp614Gly	62	241	98.41	94.14	0.30	Benign/tolerated	0.164016
C14408T	Pro4715Leu	61	234	96.83	91.41	0.31	Benign/tolerated	0.144062
G25563T	Gln57His	39	142	61.90	55.47	0.00	Deleterious	0.355651
C28854T	Ser194Leu	30	90	47.62	35.16	0.00	Deleterious	0.067355
G16078A	Val5272Ile	7	10	11.11	3.91	0.00	Deleterious	0.022562
G23311T	Glu583Asp	5	10	7.94	3.91	0.33	Benign/tolerated	0.175819
C23277T	Thr572Ile	4	5	6.35	1.95	0.57	Benign/tolerated	0.059057
G21724T	Leu54Phe	3	39	4.76	15.23	0.69	Benign/tolerated	0.027646
C18568T	Leu6102Phe	2	33	3.17	12.89	0.01	Deleterious	0.027074
A2292C	Gln676Pro	2	31	3.17	12.11	0.05	Deleterious	0.036972

**TABLE 4:** Chi-square test analysis of the deceased and recovered 490 patients for gender and age group.

		**Gujarat (*n* = 319)**		**Global (*n* = 2,121)**		
		**Deceased**	**Recovered**	**Deceased**	**Recovered**	***p*-Value**
Total sample		63	256	276	1,845	0.00118
Gender	Male	37	178	203	1,002	0.89596
	Female	26	78	73	843	2.7E-08
Age (years)	0–40	2	94	18	865	0.97648
	41–60	28	115	101	675	0.03783
	> 60	33	47	157	305	0.20849

## Discussion

India is a densely populated country and needs to tackle the challenges of the COVID-19 pandemic through management strategies and the stringent implementation of policies. The genome sequencing efforts have been enormously useful in understanding the pathogenic and adaptive behavior of viruses in the Indian population. The epidemiological approach-based method in a resource-poor setting, such as Telangana and Andhra Pradesh states, revealed that the case-fatality ratios spanned 0.05% at ages 5–17 years to 16.6% at ages ≥85 years (Laxminarayan et al., 2020). Similarly, immune response, food habits, and gut–microbiome dynamics might also play key roles in the SARS-CoV-2 viral outbreak that should further help in identifying host-related responses and better control strategies (Bajaj and Purohit, 2020; Shastri et al., 2020). Furthermore, to understand virus pathogenesis dynamics in the populations of Gujarat, genome sequencing of the SARS-COV-2 clinical positive samples was carried out. SARS-CoV-2 viral genome analysis from Gujarat highlights the distinct genomic attributes, geographical distribution, age composition, and gender classification. These features also highlight unique genomic patterns in terms of synonymous and missense variants associated with the prevalence of dominant clades and lineages with distinct geographical locations in Gujarat. Our research study highlights the most comprehensive genomic resources available so far from Gujarat, India. Therefore, identifying variants specific to the deceased and recovered patients would certainly aid in better treatment and COVID-19 containment strategy. The fatality rate compared with different geographical locations may point toward the higher virulence profile of certain viral strains with lethal genetic mutations, but this remains to be clinically unestablished. Perhaps the onset of clinical features in symptomatic patients helps in prioritizing the diagnosis and testing strategy.

The first case report of complete genome sequence information from India is from a patient in Kerala with a direct travel history to Wuhan, China. Similarly, other isolates from India cluster with Iran, Italy, Spain, England, United States, and Belgium, and probably similar isolates are transmitting in India and may have variable mutation profile (Mondal et al., 2020; Potdar et al., 2020; Yadav et al., 2020). The dominance of a particular lineage or clade at a particular location merely does not establish the biological function of the virus type isolate in terms of higher death rate but the epidemiological factors, such as clinically diagnosed co-morbidity, age, gender, or asymptomatic transmission, that are the most likely influencing factor in transmission. Sampling biases could certainly influence the prediction models, but it would narrow down to particular types of isolates and unique mutations that can further be experimentally validated to establish their clinical significance.

The geographical distribution of the viral isolates is denoted in the phylogeny with the maximum SARS-CoV-2 positive samples sequenced from Ahmedabad (*n* = 172), followed by Vadodara (*n* = 92), Surat (*n* = 86), and Gandhinagar (*n* = 30). The distribution of dominant lineages in Ahmedabad is steered by occurrences of B.1.36 (*n* = 75), B.1 (*n* = 55), and B.6 (*n* = 2). The concept of lineages, clades, haplotypes, or genotypes is slightly perplexing and overlapping in terms of definitions with respect to different repositories and analytics. Therefore, it is most important to define mutations in the isolates that determine their unique position in phylogeny in terms of geographical distribution, age, gender, and locations of the genotypes, etc. Phylogenetic distribution of the viral genomes across different geographical locations along with metadata information should help in the evaluation of the posterior distribution, virulence, divergence times, and evolutionary rates in viral populations (Drummond and Rambaut, 2007). The recurrent mutations occurring independently multiple times in the viral genomes are hallmarks of convergent evolution in viral genomes with significance in host adaptability, spread, and transmission, even though contested in terms of mechanisms driving the pathogenicity and virulence across different hosts and specifically to human populations across different geographical locations (Grifoni et al., 2020; van Dorp et al., 2020).

### Incidence of Mutations in Deceased and Recovered Patients

In the context of the globally prevalent mutations across different geographical locations, we have analyzed viral genome isolates with the most frequent mutations present in the patients from those who have suffered casualties. The higher death rate, especially in Ahmedabad, India, became a cause of serious concern and remains elusive to be identified with enough scientific evidence. We have identified differentially dominant and statistically significant mutations prevalent in the viral genome isolates in Gujarat, India.

The dominant mutations in the deceased patients represented by the change in A23403G were observed at a frequency of 98.41% in the Gujarat genomes (*p*-value of 0.1640) and 74.28% in the global genomes with known patient status (*p*-value of 0.5223). These missense mutations are found to be observed in the spike protein of the SARS-CoV-2 genome. The well-known function of the viral spike protein is in mediating the infection by interacting with the angiotensin-converting enzyme 2 (ACE2) receptor (Li et al., 2005; Chu et al., 2020; Guan et al., 2020; Guo et al., 2020) of the human host species. Another mutation, C14408T with a frequency of 96.83%, is present in the Orf1b gene encoding RNA-directed RNA polymerase (RDRP) non-structural protein (nsp12) with a *p*-value of 0.1440 in deceased versus recovered patients from Gujarat, while also being observed to be statistically significant in the global dataset with a *p*-value of 8.28E-05 with a frequency of 88.77%. The comparative analysis of the deceased patients (*n* = 63) and recovered patients (*n* = 256) in Gujarat as highlighted in Figure 6 is represented by a Venn diagram. In contrast, the functional role of the RDRP enzyme activity is necessary for the viral genome replication and transcription of most RNA viruses (Imbert et al., 2006; Velazquez-Salinas et al., 2020). The MNV GGG28881AAC that is a missense mutation with a change in ArgGly203LysArg in the N gene is a deleterious mutation and is dominant in global viral genomes with a frequency in deceased (39.45%) and recovered patients (31.38%).

The exclusive dominant mutations present in the population of Gujarat were G25563T and C28854T in the Orf3a and N genes, respectively. The Orf3a gene encodes a protein involved in the regulation of inflammation, antiviral responses, and apoptosis. Mutation in these regions alters the functional profile of the nuclear factor-kβ (NF-kβ) activation and nucleotide-binding domain leucine-rich repeat containing (NLRP3) inflammasome. One of the main features of Orf3a protein is having the presence of a cysteine-rich domain, which participates in the enzymatic nucleophilic substitution reactions. This protein is expressed abundantly in infected and transfected cells, which localizes to the intracellular and plasma membranes and induces apoptosis in transfected and infected cells (Issa et al., 2020). This enzyme mediates the extensive proteolytic processing of two overlapping replicase polyproteins, pp1a and pp1ab, to yield the corresponding functional polypeptides that are essential for coronavirus replication and transcription processes (Kohlmeier and Woodland, 2009; Benvenuto et al., 2020). While in the case of mutation at position C28311T leading to change of amino acid proline to leucine, the enzyme lies in the N gene that has a role in virion assembly and release and plays a significant role in the formation of replication–transcription complexes (Alsaadi and Jones, 2019; Liu, 2019; Wu et al., 2020; Yin, 2020). Similarly, the N protein is a highly basic protein that could modulate viral RNA synthesis (Millet and Whittaker, 2015; Hassan et al., 2020; Sarif Hassan et al., 2020).

**FIGURE 7:**
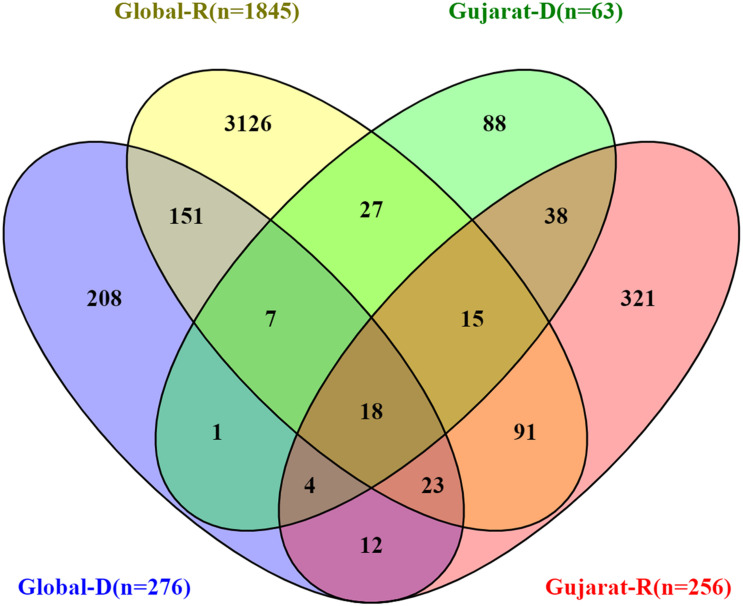
Overall comparison of the missense mutations in SARS-CoV-2 genome. Gujarat (R = 256, D = 63) and Global (R = 1,845, D = 276), where “R” is the number of genomes from recovered patients, and “D” is the number of genomes from deceased patients.

The SIFT scores of these mutations were determined and also signify the functional effect change in whether an amino acid substitution affects protein function or not in terms of the deleterious effect or benign tolerated (Vaser et al., 2016). The predicted SIFT score of the mutation G25563T in the Orf3a and C28854T in the N gene was classified to be deleterious in nature. Similarly, a comparison analysis of the global (*n* = 79,518), India (*n* = 1,821), and Gujarat datasets (*n* = 502), where the “*n*” is the number of genomes included in the analysis, indicates the overall dominance of C241T, C3037T, A23403G, C14408T, and G25563T. Furthermore, it is suggestive of the comparative dominant mutation profile, including non-synonymous and missense mutations. The analysis of the dataset from the global deceased (*n* = 276) and recovered patients (*n* = 1,845) with known status from the metadata information available on the GISAID server with the complete genome sequences considered in the analysis indicates the dominance of the missense mutations at A23403G, C14408T, C1059T, and G25563T. The overall comparison of the mutation profile of the patient dataset of deceased and recovered samples is highlighted in Figure 7, from global and Gujarat.

**FIGURE 8:**
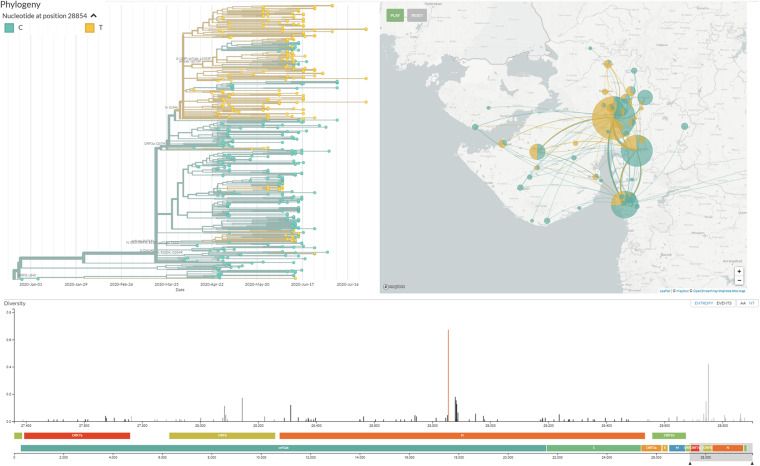
Distinct cluster of the viral isolate with mutation C28854T/Ser194Leu/N gene in Gujarat SARS-CoV-2 genomes. This cluster is visualized at http://covid.gbrc.org.in/nextstrain.php using the Nextstrain virus genome analysis pipeline.

Mutation in the N gene at C28854T and mutation in the Orf3a gene at G25563T were found to be dominant among deceased patients from Gujarat. Moreover, C28854T is forming a distinct sub-cluster under 20A (A2a as per the old classification of the next strain) clade, highlighted in Figure 8. The same is proposed as a new sub-clade 20D in the next strain and GHJ in GISAID. This sub-clade is also present in genomes sequenced from Bangladesh and Saudi Arabia. Both these proteins play significant roles in viral replication and pathogenesis (Luan et al., 2020; Pachetti et al., 2020; Peter and Schug, 2020). The association of the mutations with the viral transmission and mortality rate remains a mystifying puzzle for the global scientific community. The identification and validation of these mutations should pave the way forward for the development of treatment and diagnostics of coronavirus disease. The evading host immune response and defense mechanism sufficiently improve the adaptive behavior of the pathogenic species, thus making them highly contagious. Further, laboratory and experimental studies need to be carried out to validate the exact role of this particular mutation with respect to the molecular pathways and interactions in the biological systems despite being a strong possible mutation candidate found in the Gujarat region.

The genomics-based approach has been a useful resource to identify and characterize virulence, pathogenicity, and host adaptability. Further, identification and characterization of the frequently mutated positions in the SARS-CoV-2 genome will certainly help in the deeper understanding of the infection biology of coronaviruses, development of vaccines and therapeutics, and potential drug repurpose candidates using predictive computational biology and experimental validations. The present study highlights the genome sequencing, haplotyping, and mutation profile of the 502 SARS-CoV-2 viral genome isolates from 46 different locations representing 20 districts across Gujarat, India. Furthermore, we have reported significant variants associated with mortality in the Gujarat and global viral genomes. As the pandemic is progressing, the virus is also diverging into different clades. This also provides adaptive advantages to viruses in the progression of the disease and its pandemic potential. In this study, we have reported a distinct cluster of coronavirus under 20A clade of Nextstrain and proposed it as 20D as per the next strain analysis or GHJ as per the GISAID analysis, predominantly present in the Gujarat genomes. Understanding the SARS-CoV-2 genome and tracking its evolution will help in devising better strategies for the development of diagnosis, treatment, and vaccine candidates in response to the global pandemic.

## Data Availability Statement

The raw data generated in this study have been submitted to the NCBI BioProject (https://www.ncbi.nlm.nih.gov/bioproject) under accession number PRJNA625669. Supplementary Dataset to this manuscript are also available at Mendeley Data with DOI: 10.17632/pc38m6mwxt.1 (https://data.mendeley.com/datasets/pc38m6mwxt/draft?a=1aa66c2a-5b93-456f-816c-3f26a4 82dc2a). All datasets of COVID-19 are also provided on GBRC-COVID portal (http://covid.gbrc.org.in/).

## Ethics Statement

The studies involving human participants were reviewed and approved by Institutional Ethical Committee of GBRC and respective government medical colleges. Gujarat Biotechnology Research Centre, Gandhinagar B.J. Medical College and Civil hospital, Ahmedabad Banas Medical College and Research Institute, Palanpur Department of MicroBiology, Government Medical College, Surat Dr. N. D. Desai Medical College & Hospital, Nadiad Dr. RSS Hospital, Modasa GAIMS & G K General Hospital, Bhuj GMERS Medical College and Hospital, Gandhinagar GMERS Medical College and Hospital, Gotri, Vadodara GMERS Medical College and Hospital, Himmatnagar Government Medical College, Bhavnagar Government Medical College, Vadodara Pandit Deendayal Upadhyay Government Medical College, Rajkot Saikrishna Hospital, Mehsana Sardar Vallabhbhai Patel Institute of Medical Sciences & Research. Written informed consent to participate in this study was provided by the participants’ legal guardian/next of kin.

## Author Contributions

MJ, SB, and CJ conceptualized the work plan and guided it for analysis of primary data, interpretation of data, and editing of the manuscript. AP, DK, AA, and MJ retrieved and analyzed the data and generated the tables and figures under supervision of CJ. MJ, DK, and AP wrote the manuscript. MP, JR, ZP, PT, and MG did the sample processing and RNA isolation. LP, KP, and NS did the genome sequencing. SK did the data analysis and manuscript editing. All authors contributed to the article and approved the submitted version.

## Conflict of Interest

The authors declare that the research was conducted in the absence of any commercial or financial relationships that could be construed as a potential conflict of interest.

## Footnotes

1 https://www.gisaid.org/

2 https://github.com/nextstrain/augur

## References

[B1] Alsaadi E. A. J. Jones I. M. (2019). Membrane binding proteins of coronaviruses. *Future Virol.* 14 275–286. 10.2217/fvl-2018-2144 32201500 PMC7079996

[B2] Andrews S. (2010). *FastQC: a Quality Control Tool for High Throughput Sequence Data.* Babraham: Babraham institute.

[B3] Bajaj A. Purohit H. J. (2020). Understanding SARS-CoV-2: genetic diversity, transmission and cure in human. *Indian J. Microbiol.* 60 1–4. 10.1007/s12088-020-00869-864 32317810 PMC7169643

[B4] Benvenuto D. Angeletti S. Giovanetti M. Bianchi M. Pascarella S. Cauda R. (2020). Evolutionary analysis of SARS-CoV-2: how mutation of non-structural protein 6 (NSP6) could affect viral autophagy. *J. Infect.* 81 e24–e27. 10.1016/j.jinf.2020.03.058 32283146 PMC7195303

[B5] Chu D. K. W. Pan Y. Cheng S. M. S. Hui K. P. Y. Krishnan P. Liu Y. (2020). Molecular diagnosis of a novel coronavirus (2019-nCoV) causing an outbreak of pneumonia. *Clin. Chem.* 555 549–555. 10.1093/clinchem/hvaa029 32031583 PMC7108203

[B6] Drummond A. J. Rambaut A. (2007). BEAST: bayesian evolutionary analysis by sampling trees. *BMC Evol. Biol.* 7:214. 10.1186/1471-2148-7-214 17996036 PMC2247476

[B7] Du L. He Y. Zhou Y. Liu S. Zheng B. J. Jiang S. (2009). The spike protein of SARS-CoV - a target for vaccine and therapeutic development. *Nat. Rev. Microbiol.* 7 226–236. 10.1038/nrmicro2090 19198616 PMC2750777

[B8] Evenett S. J. Winters L. A. (2020). *Preparing for a Second Wave of Covid-19: A Trade Bargain to Secure Supplies of Medical Goods*. Global Trade Alert. Available online at: https://blogs.sussex.ac.uk/uktpo/publications/preparing-for-a-second-wave-of-covid-19-a-trade-bargain-to-secure-supplies-of-medical-goods/ (accessed July 20, 2020).

[B9] Grifoni A. Sidney J. Zhang Y. Scheuermann R. H. Peters B. Sette A. (2020). A sequence homology and bioinformatic approach can predict candidate targets for immune responses to SARS-CoV-2. *Cell Host Microbe* 27 671—–680.e2. 10.1016/j.chom.2020.03.002 32183941 PMC7142693

[B10] Guan W. Ni Z. Hu Y. Liang W. Ou C. He J. (2020). Clinical characteristics of coronavirus disease 2019 in China. *N. Engl. J. Med.* 382 1708–1720. 10.1056/NEJMoa2002032 32109013 PMC7092819

[B11] Guo Y. R. Cao Q. D. Hong Z. S. Tan Y. Y. Chen S. D. Jin H. J. (2020). The origin, transmission and clinical therapies on coronavirus disease 2019 (COVID-19) outbreak- a n update on the status. *Mil. Med. Res.* 7:11. 10.1186/s40779-020-00240-0 32169119 PMC7068984

[B12] Hadfield J. Megill C. Bell S. M. Huddleston J. Potter B. Callender C. (2018). NextStrain: real-time tracking of pathogen evolution. *Bioinformatics* 34 4121–4123. 10.1093/bioinformatics/bty407 29790939 PMC6247931

[B13] Hassan S. S. Choudhury P. P. Basu P. Jana S. S. (2020). Molecular conservation and differential mutation on ORF3a gene in Indian SARS-CoV2 genomes. *Genomics* 112 3226–3237. 10.1016/j.ygeno.2020.06.016 32540495 PMC7291963

[B14] Imbert I. Guillemot J. C. Bourhis J. M. Bussetta C. Coutard B. Egloff M. P. (2006). A second, non-canonical RNA-dependent RNA polymerase in SARS coronavirus. *EMBO J.* 25 4933–4942. 10.1038/sj.emboj.7601368 17024178 PMC1618104

[B15] Issa E. Merhi G. Panossian B. Salloum T. Tokajian S. (2020). SARS-CoV-2 and ORF3a: nonsynonymous mutations, functional domains, and viral pathogenesis. *mSystems* 5:e00266-20. 10.1128/msystems.00266-220 PMC7205519 32371472

[B16] Katoh K. Standley D. M. (2013). MAFFT multiple sequence alignment software version 7: improvements in performance and usability. *Mol. Biol. Evol.* 30 772–780. 10.1093/molbev/mst010 23329690 PMC3603318

[B17] Kohlmeier J. E. Woodland D. L. (2009). Immunity to respiratory viruses. *Annu. Rev. Immunol.* 27 61–82. 10.1146/annurev.immunol.021908.132625 18954284

[B18] Laxminarayan R. Wahl B. Dudala S. R. Gopal K. Mohan B. C. Neelima S. (2020). Epidemiology and transmission dynamics of COVID-19 in two Indian states. *Science* 370 691–697. 10.1126/science.abd7672 33154136 PMC7857399

[B19] Leung K. Wu J. T. Liu D. Leung G. M. (2020). First-wave COVID-19 transmissibility and severity in China outside Hubei after control measures, and second-wave scenario planning: a modelling impact assessment. *Lancet* 395 1382–1393. 10.1016/S0140-6736(20)30746-30747 32277878 PMC7195331

[B20] Li W. Zhang C. Sui J. Kuhn J. H. Moore M. J. Luo S. (2005). Receptor and viral determinants of SARS-coronavirus adaptation to human ACE2. *EMBO J.* 24 1634–1643. 10.1038/sj.emboj.7600640 15791205 PMC1142572

[B21] Liu D. X. (2019). Human coronavirus: host-pathogen interaction. *Annu. Rev. Microbiol.* 73 529–557. 10.1146/annurev-micro-020518 31226023

[B22] Luan J. Lu Y. Jin X. Zhang L. (2020). Spike protein recognition of mammalian ACE2 predicts the host range and an optimized ACE2 for SARS-CoV-2 infection. *Biochem. Biophys. Res. Commun.* 526 165–169. 10.1016/j.bbrc.2020.03.047 32201080 PMC7102515

[B23] Mercatelli D. Giorgi F. M. (2020). Geographic and genomic distribution of SARS-CoV-2 mutations. *Front. Microbiol.* 11:800. 10.3389/fmicb.2020.01800 32793182 PMC7387429

[B24] Millet J. K. Whittaker G. R. (2015). Host cell proteases: critical determinants of coronavirus tropism and pathogenesis. *Virus Res.* 202 120–134. 10.1016/j.virusres.2014.11.021 25445340 PMC4465284

[B25] Minh B. Q. Schmidt H. A. Chernomor O. Schrempf D. Woodhams M. D. von Haeseler A. (2020). IQ-TREE 2: new models and efficient methods for phylogenetic inference in the genomic era. *Mol. Biol. Evol.* 37 1530–1534. 10.1093/molbev/msaa015 32011700 PMC7182206

[B26] Mondal M. Lawarde A. Somasundaram K. (2020). Genomics of Indian SARS-CoV-2: implications in genetic diversity, possible origin and spread of virus. *Medrxiv* [preprint] 10.1101/2020.04.25.20079475

[B27] Pachetti M. Marini B. Benedetti F. Giudici F. Mauro E. Storici P. (2020). Emerging SARS-CoV-2 mutation hot spots include a novel RNA-dependent-RNA polymerase variant. *J. Transl. Med.* 18:179. 10.1186/s12967-020-02344-6 32321524 PMC7174922

[B28] Peter E. K. Schug A. (2020). The inhibitory effect of a Corona virus spike protein fragment with ACE2. *bioRxiv* [preprint] 10.1101/2020.06.03.132506 PMC7451127 32941783

[B29] Potdar V. Cherian S. Deshpande G. Ullas P. Yadav P. Choudhary M. (2020). Genomic analysis of SARS-CoV-2 strains among Indians returning from Italy, Iran & China, & Italian tourists in India. *Indian J. Med. Res.* 151 255–260. 10.4103/ijmr.IJMR_1058_20 32362650 PMC7366550

[B30] Rambaut A. (2018). *FigTree 1.4.4 Software.* Edinburgh: Institute of Evolutionary Biology University.

[B31] Sagulenko P. Puller V. Neher R. A. (2018). TreeTime: maximum-likelihood phylodynamic analysis. *Virus Evol.* 4:vex042. 10.1093/ve/vex042 29340210 PMC5758920

[B32] Sarif Hassan S. Pal Choudhury P. Roy B. Sankar Jana S. (2020). Missense mutations in SARS-CoV2 genomes from Indian patients. *Genomics* 112 4622–4627. 32822756 10.1016/j.ygeno.2020.08.021 PMC7434617

[B33] Schmieder R. Edwards R. (2011). Quality control and preprocessing of metagenomic datasets. *Bioinformatics* 27 863–864. 10.1093/bioinformatics/btr026 21278185 PMC3051327

[B34] Shastri A. Wheat J. Agrawal S. Chaterjee N. Pradhan K. Goldfinger M. (2020). Delayed clearance of SARS-CoV2 in male compared to female patients: high ACE2 expression in testes suggests possible existence of gender-specific viral reservoirs. *medRxiv* [preprint] 10.1101/2020.04.16.20060566

[B35] Shereen M. A. Khan S. Kazmi A. Bashir N. Siddique R. (2020). COVID-19 infection: origin, transmission, and characteristics of human coronaviruses. *J. Adv. Res.* 24 91–98. 10.1016/j.jare.2020.03.005 32257431 PMC7113610

[B36] Strzelecki A. (2020). The second worldwide wave of interest in coronavirus since the COVID-19 outbreaks in South Korea, Italy and Iran: a google trends study. *Brain. Behav. Immun.* 88 950–951. 10.1016/j.bbi.2020.04.042 32311493 PMC7165085

[B37] van Dorp L. Acman M. Richard D. Shaw L. P. Ford C. E. Ormond L. (2020). Emergence of genomic diversity and recurrent mutations in SARS-CoV-2. *Infect. Genet. Evol.* 83:104351. 10.1016/j.meegid.2020.104351 32387564 PMC7199730

[B38] Vaser R. Adusumalli S. Leng S. N. Sikic M. Ng P. C. (2016). SIFT missense predictions for genomes. *Nat. Protoc.* 11 1–9. 10.1038/nprot.2015.123 26633127

[B39] Velazquez-Salinas L. Zarate S. Eberl S. Novella I. Borca M. V. (2020). Positive selection of ORF3a and ORF8 genes drives the evolution of SARS-CoV-2 during the 2020 COVID-19 pandemic. *bioRxiv* [preprint] 10.1101/2020.04.10.035964 PMC7644918 33193132

[B40] Wu A. Peng Y. Huang B. Ding X. Wang X. Niu P. (2020). Genome composition and divergence of the novel coronavirus (2019-nCoV) originating in China. *Cell Host Microbe* 27 325–328. 10.1016/j.chom.2020.02.001 32035028 PMC7154514

[B41] Xu S. Li Y. (2020). Beware of the second wave of COVID-19. *Lancet* 395 1321–1322. 10.1016/S0140-6736(20)30845-X 32277876 PMC7194658

[B42] Yadav P. Potdar V. Choudhary M. Nyayanit D. Agrawal M. Jadhav S. (2020). Full-genome sequences of the first two SARS-CoV-2 viruses from India. *Indian J. Med. Res.* 151 200–209. 10.4103/ijmr.IJMR_663_20 32242873 PMC7258756

[B43] Yin C. (2020). Genotyping coronavirus SARS-CoV-2: methods and implications. *Genomics* 112 3588–3596. 10.1016/j.ygeno.2020.04.016 32353474 PMC7184998

[B44] Zhang D. Gao F. Jakovliæ I. Zou H. Zhang J. Li W. X. (2020). PhyloSuite: an integrated and scalable desktop platform for streamlined molecular sequence data management and evolutionary phylogenetics studies. *Mol. Ecol. Resour.* 20 348–355. 10.1111/1755-0998.1 31599058

