# Supplementary material for: Genomic Variations in SARS-CoV-2 Genomes From Gujarat: Underlying Role of Variants in Disease Epidemiology
Source: Front Genet. 2021 Mar 19;12:586569. doi: 10.3389/fgene.2021.586569 (PMC8017293; doi:10.3389/fgene.2021.586569)
Supplement: Supplementary file 1 [file Data_Sheet_1.PDF]

This dataset represents additional information manuscript entitled “Genomic variations in SARS-CoV-2 genomes from Gujarat: Underlying role of variants in disease epidemiology”

Supplementary Table S1: The details of the samples collected from each location along with districts from the Gujarat State

Location	District	No of Genomes
Ahmedabad	Ahmedabad	166
Daskroi	Ahmedabad	5
Dholka	Ahmedabad	1
Bayad	Aravalli	1
Dhansura	Aravalli	1
Modasa	Aravalli	20
Dhanera	Banaskantha	1
Palanpur	Banaskantha	6
Bharuch	Bharuch	1
Bhavnagar	Bhavnagar	3
Ghogha	Bhavnagar	1
Mahuva	Bhavnagar	1
Botad	Botad	2
Dahod	Dahod	5
Dahegam	Gandhinagar	4
Gandhinagar	Gandhinagar	19
Kalol	Gandhinagar	5
Mansa	Gandhinagar	2
Kodinar	Gir Somnath	1
Una	Gir Somnath	3
Jamnagar	Jamnagar	6
Junagadh	Junagadh	3
Kapadvanj	Kheda	1
Kheda	Kheda	4
Mahemdavad	Kheda	2
Nadiad	Kheda	3
Bhuj	Kutch	1
Mandvi	Kutch	3
Kadi	Mahesana	2
Mehsana	Mehsana	3
Gondal	Rajkot	2
Jetpur	Rajkot	2
Rajkot	Rajkot	22
Himatnagar	Sabarkantha	8
Khedbrahma	Sabarkantha	3
Prantij	Sabarkantha	8
Talod	Sabarkantha	1
Adajan	Surat	2
Bardoli	Surat	2
Choryasi	Surat	11
Kamrej	Surat	2
Olpad	Surat	2
Surat	Surat	67
Chuda	Surendranagar	2
Savli	Vadodara	4
Vadodara	Vadodara	88
		502
