# Supplementary material for: Genomic Variations in SARS-CoV-2 Genomes From Gujarat: Underlying Role of Variants in Disease Epidemiology
Source: Front Genet. 2021 Mar 19;12:586569. doi: 10.3389/fgene.2021.586569 (PMC8017293; doi:10.3389/fgene.2021.586569)
Supplement: Supplementary file 2 [file Data_Sheet_2.PDF]

This dataset represents additional information manuscript entitled “Genomic variations in SARS-CoV-2 genomes from Gujarat: Underlying role of variants in disease epidemiology”

Supplementary Table S3: Lineage and Clade distribution of SARS-CoV-2 from the Districts of the Gujarat State

**District**	**Clade**						**Lineage**									
	**G**	**GH**	**GR**	**L**	**O**	**S**	**A**	**B**	**B.1**	**B.1.1**	**B.1.1.32**	**B.1.113**	**B.1.36**	**B.1.80**	**B.6**	**B.6**
Ahmedabad	54	115	1	1	1				55	1		38	75	1	2	2
Aravalli	1	21							1				21			
Banaskantha	3	4							1	1			5			
Bharuch	1								1							
Bhavnagar	1	3	1						1		1	2	1			
Botad		1			1								1		1	1
Dahod	1				4				1						4	4
Gandhinagar	14	16							14			2	14			
Gir Somnath		1	2		1					2			1		1	1
Jamnagar	1	4			1				1				4		1	1
Junagadh	3								3							
Kheda	2	7				1	1		2			1	6			
Kutch	1	2	1						1	1		1	1			
Mahesana	2								2							
Mehsana		3										3				
Rajkot	6	15				5	5		6				15			
Sabarkantha	9	10			1			1	10			2	7			
Surat	37	27	1		9	12	12	3	41	1		1	25		3	3
Surendranagar		2											2			
Vadodara	44	47	1						42	3		11	36			
**Grand Total**	**180**	**278**	**7**	**1**	**18**	**18**	**18**	**4**	**182**	**9**	**1**	**61**	**214**	**1**	**12**	**12**
